# Expression Profile of Selected Genes Involved in Storage Lipid Synthesis in a Model Oleaginous Yeast Species *Yarrowia lipolytica*

**DOI:** 10.3390/ijms23031041

**Published:** 2022-01-18

**Authors:** Agata Fabiszewska, Magdalena Paplińska-Goryca, Paulina Misiukiewicz-Stępień, Małgorzata Wołoszynowska, Dorota Nowak, Bartłomiej Zieniuk

**Affiliations:** 1 Department of Chemistry, Institute of Food Sciences, Warsaw University of Life Sciences-SGGW, 159c Nowoursynowska Street, 02-776 Warsaw, Poland; bartlomiej_zieniuk@sggw.edu.pl; 2 Department of Internal Medicine, Pulmonary Diseases and Allergy, Medical University of Warsaw, 1a Banacha Street, 02-097 Warsaw, Poland; mpaplinska@wum.edu.pl (M.P.-G.); pmisiukiewicz@wum.edu.pl (P.M.-S.); 3 Postgraduate School of Molecular Medicine, Medical University of Warsaw, 2a Trojdena Street, 02-091 Warsaw, Poland; 4 Łukasiewicz Research Network—Institute of Industrial Organic Chemistry, 6 Annopol Street, 03-236 Warsaw, Poland; malgorzata.woloszynowska@ipo.lukasiewicz.gov.pl; 5 Department of Food Engineering and Process Management, Institute of Food Sciences, Warsaw University of Life Sciences-SGGW, Nowoursynowska Street 159c, 02-776 Warsaw, Poland; dorota_nowak@sggw.edu.pl

**Keywords:** ex novo lipid biosynthesis, lipid metabolism, oleaginous yeast, single cell oil, *Yarrowia lipolytica*

## Abstract

*Yarrowia lipolytica* yeast is a model species of the group of oleaginous microorganisms capable of intracellular lipids accumulation in an amount exceeding 20% of the dry mass. Single cell oil biosynthesis can follow one of two biochemical pathways—de novo accumulation of cellular lipids in medium containing non-lipid carbon sources (including saccharides, glycerol) and ex novo microbial oil synthesis which involves fatty acids uptake from the environment. The mRNA expression of selected genes of de novo and ex novo lipid synthesis pathways was analyzed and correlated with the phenotypically observed features. It was proved that the accumulation yield of storage lipids via ex novo pathway was to some extent dependent on the limitation of the nitrogen source in the medium. It was also proposed that the synthesis of intracellular lipids in lipid-rich medium proceeded mainly via ex novo pathway, although the activity of genes encoding the enzymes of the de novo pathway were not completely inhibited at the stage of transcription by fatty acids present in the medium (e.g., ATP-citrate lyase). Molecular markers of two biosynthesis routes has been outlined and a hypothetical connection point between de novo and ex novo route were indicated.

## 1. Introduction

Restricted number of microorganisms have been recorded to be capable of growing on fats and at the same time accumulate significant lipid quantities [1]. *Y. lipolytica* yeast is a model species of the group of both lipolytic and oleaginous microorganisms accumulating lipids in an amount exceeding 20% of the cell dry mass. The main storage lipids are triacylglycerides (TAG, 90%) and in a lesser extent steryl esters (SE). These neutral lipids are being accumulated into a specialized organelle of the cell called the lipid body (LB) [2,3]. Utilization of microbial oils as a substrate for biofuel production and its application in food and feed industry represents a promising way for replacement of plant oils in the energy and food sector in light of declining earth aerals and climate change [4,5]. Enhancing the single cell oil production of *Y. lipolytica* requires biotechnological modifications to increase its ability to store lipids which can be achieved by altering cultivation parameters or through genetic manipulation [6]. Research on the role of enzymes and its regulation for model *Y. lipolytica* yeasts is generating considerable interest in terms of synthetic biology and its practical soundness. Notably, in contrast to animals and plants, microorganisms can be easily engineered and accepted by the society as the safety of this yeast has been recently assessed [7].

**Figure 1:**
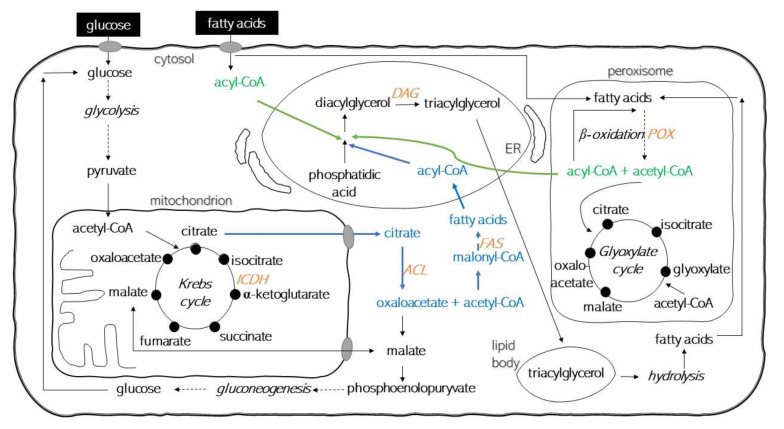
Overview of de novo (blue color) and ex novo (green color) pathways involved in lipid metabolism and their link with glyoxylate and the TCA cycles (in yeast cell?). The dashed line represents the multistep process. Orange abbreviations represent genes encoding key enzymes investigated in the study: *ACL*—ATP-citrate lyase, *FAS*—fatty acid synthase, *ICDH*—NADP-dependent isocitrate dehydrogenase, *DAG*—diacylglycerol acyltransferase, *POX*—acyl-coenzyme A oxidase (adopted and modified after [1,9]).

The storage lipid biosynthesis may follow one of two biochemical pathways (Figure 1). The de novo synthesis is described by synthesis of fatty acid precursors and their inclusion in the path of storage lipid synthesis and it takes place in medium containing non-lipid carbon sources (including saccharides or glycerol). Fatty acids are produced in the cytosol of the *Y. lipolytica* yeast cells from the building block acetyl-CoA, which can come from ATP citrate lyase action. *ACL* genes have been considered a hallmark of oleaginous microorganisms and are not present in non-oleaginous species [6,7]. The fatty acid synthase (FAS), a second key-enzyme in the de novo pathway produces acyl-CoA as an initiation molecule and malonyl-CoA as elongation unit (Figure 1). Activated 16:0 and 18:0 molecules are substrates for elongases and desaturases to produce long chain fatty acids and unsaturated fatty acids via Kennedy pathway [7,8].

The ex novo route is characterized by using fatty acids, partially or by completing their metabolism by β-oxidation and accumulation in lipid bodies. In the cytosol, free fatty acids can be activated by fatty acyl-CoA synthetase to produce acyl-CoA [10]. It is believed that ex novo synthesis of microbial oil occurs only in media with lipid carbon sources [1]. Meanwhile, the results of preliminary studies allowed for the formulation of a hypothesis that in media containing lipid carbon sources, the synthesis of storage lipids could take place in parallel with the activation of two pathways [11].

Previous work was provided in nitrogen-limited glucose or olive oil-rich media and one control was chosen in YPG medium. The preliminary study has only focused on transcription level of two genes: *POX2* which encodes acyl-CoA oxidase II involved in β-oxidation of fatty acids and *ACL* which encoded ATP-citrate lyase—enzyme crucial for de novo lipid biosynthesis [11]. The aim of the present study was to verify the hypothesis concerning the possibility of simultaneous biosynthesis of storage lipids in *Y. lipolytica* yeast cells via de novo and ex novo pathways in media containing sole lipid carbon source. Our experimental set up was based on batch cultures of the wild yeast strain *Y. lipolytica* KKP 379 carried out in a laboratory bioreactor in control media (rich in carbon and nitrogen source: YPG containing glucose, YPO containing olive oil) and in experimental media stimulating the synthesis of storage lipids (with a limited content of the nitrogen source containing 8% glucose, MG8 and 5% olive oil, MO5 and with slightly limited nitrogen source, doubled in comparison to MO5 medium and containing 5% olive oil, MO5-2xN). Moreover, the research focused on seeking genetic markers of those two biochemical routes among five selected genes encoding key enzymes of lipid biosynthesis pathways.

## 2. Results

### 2.1. Phenotypic Observations during Batch Cultures

**Figure 2:**
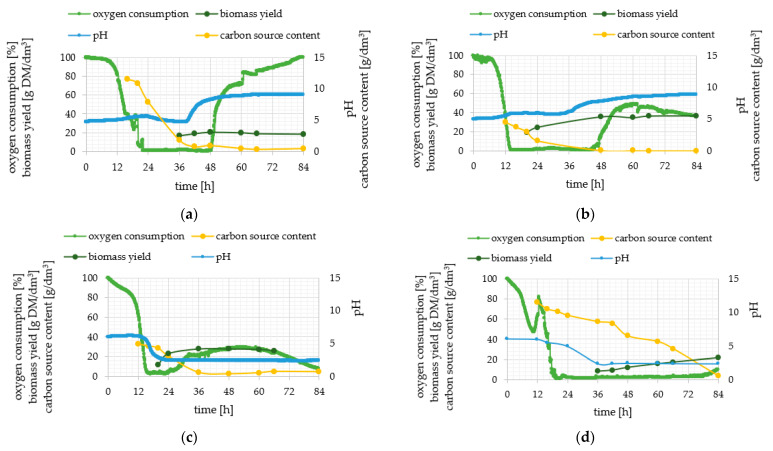
Changes in pH (thick light line), relative oxygen consumption (thick dark line), biomass yield (thin dark line) and carbon source content (thin light line; glucose or olive oil) during batch cultures of *Y. lipolytica* KKP 379 strain in YPG control (**a**), YPO control (**b**), MO5 (**c**) and MG8 (**d**) medium.

**Figure 3:**
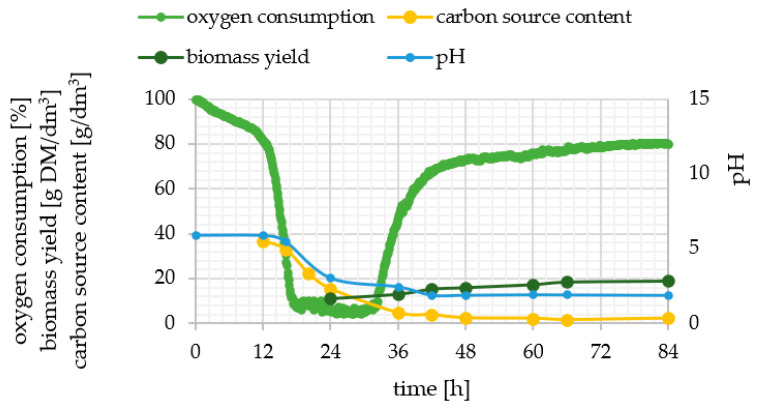
Changes in pH (thick light line), relative oxygen consumption (thick dark line), biomass yield (thin dark line) and carbon source content (thin light line; glucose or olive oil) during batch cultures of *Y. lipolytica* KKP 379 strain in MO5-2xN medium.

Two groups of genes can be distinguished: constitutive, which are described as always “on”, and genes unaffected by environmental conditions. Notably, the same conditions impact inducible gene expression in different way. The five investigated genes of de novo and ex novo lipid synthesis pathways were assigned to this second group, so that a thorough analysis of the culture process has been provided in order to correlate the phenotypically observed features with the level of genes expression (Figure 2 and Figure 3). Four culture parameters were monitored throughout all experiments—pH, oxygen consumption, biomass yield and carbon substrate content.

Five variants of cultures were analyzed and compared (Figure 2 and Figure 3). The dynamics of yeast cells growth was different because of the variety of carbon sources (glucose and olive oil) and concentration of nitrogen (limited in experimental media and rich in control ones). Media were inoculated with a small number of cells and following inoculation the lag phase lasted 12 h in YPO medium, 15 h in YPG medium and 18 h for experimental media. The log phase lasted 18 h for both media containing olive oil and 36 h for control media. Unexpectedly, no stationary phase was observed for culture performed in MG8 where an increase in biomass yield was still observed until 84 h. What should be noted is that in MO5 culture, also in the stationary phase, cells required a lot of oxygen and after 66 h the oxygen consumption decreased once again. It was symptomatic for all cultures in nitrogen-limited media that the sharp decrease in pH started at the early log phase and resulted in the low pH level at the stationary phase (final levels of pH 2.35, 2.40 and 1.91 were reached at 24, 36 and 42 h for MO5, MG8 and MO5-2xN, respectively). As was expected, control media were characterized by alkaline pH. The highest biomass yield reaching 36.7 g DM/dm^3^ was measured in YPO experiment and at 36 h there was almost no carbon source left in the medium. Comparing to YPO control medium, the carbon source was depleted at 48 h for YPG medium. Similar biomass yield at 84 h was determined in YPG (18.7 g DM/dm^3^) and MO5-2xN (18.9 g DM/dm^3^). High substrate utilization was observed in MO5 medium, because it was depleted in 36 h of culture. Similarly, in MO5-2xN medium only 4 g/dm^3^ olive oil left at 36 h and another 2 g/dm^3^ were used until the end of the experiment. The variant of the research provided in MG8 medium varied from others. Carbon source was utilized systematically during the whole period of growth and the biomass yield grew systematically during this time.

**Figure 4:**
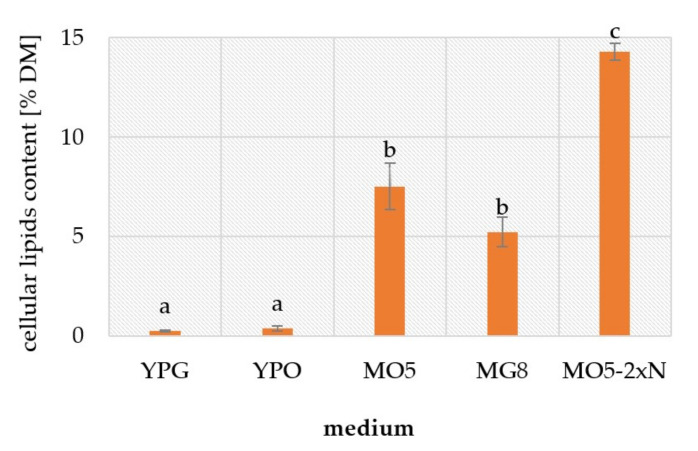
Cellular lipids content in yeast cells in the stationary phase in 84 h batch culture. The values with the same letter in a column did not differ significantly (α = 0.05). The error bars indicate standard deviation.

Referring to the final cellular lipids content (Figure 4), after 84 h of yeast culture, it can be concluded that in control YPG and YPO medium the concentration of lipids was low (0.025 and 0.036 g/g DM, respectively) because in rich medium yeast cells did not synthesize storage lipids. Significantly higher amounts were extracted from cell cultured in MO5 and MG8 media. Although the difference was not statistically relevant, results show that the content of lipid in MO5 medium was higher by 50% of the lipid amount determined for cells from MG8 culture. Noticeably, the cells derived from MO5-2xN were characterized by the highest cellular lipids concentration (0.14 g/g DM), twice as high in comparison to the medium MO5 which consisted of a twice higher C/N ratio.

**Figure 5:**
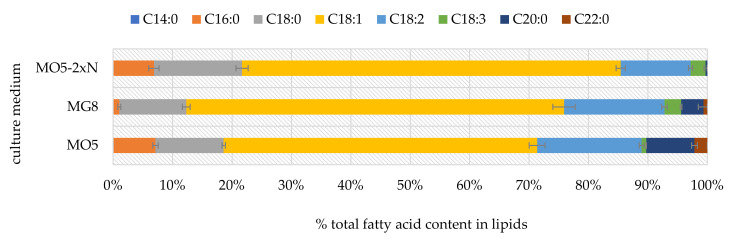
Fatty acid content in cellular lipids synthesized in MG8, MO5 and MO5-2xN media at 84th h of *Y. lipolytica* yeast culture in stationary growth phase. The error bars indicate standard deviation.

To enable the impact of carbon source on the fatty acid profile of intracellular lipids, the fatty acid contents in cellular lipids synthesized in glucose-based medium (MG8) and olive oil-based media (MO5 and MO5-2xN) were set together (Figure 5). With a few exceptions, our results show that the carbon source did not affect the examined feature. The main fatty acid present in yeast cells was oleic acid (from 52.73% for MO5 medium to 63.73% for MO5-2xN medium). In addition, high concentrations of linoleic acids were also determined (from 11.76% for cells cultured in MO5-2xN medium to 17.57% for cells cultured in MO5 medium). The sum of unsaturated fatty acids varied from 71.10% according to experiments provided in MO5 medium to 83.28% for experiments in MG8. Especially high levels of saturated C20:0 and C22:0 fatty acids were analyzed in samples from MO5-2xN medium.

**Figure 6:**
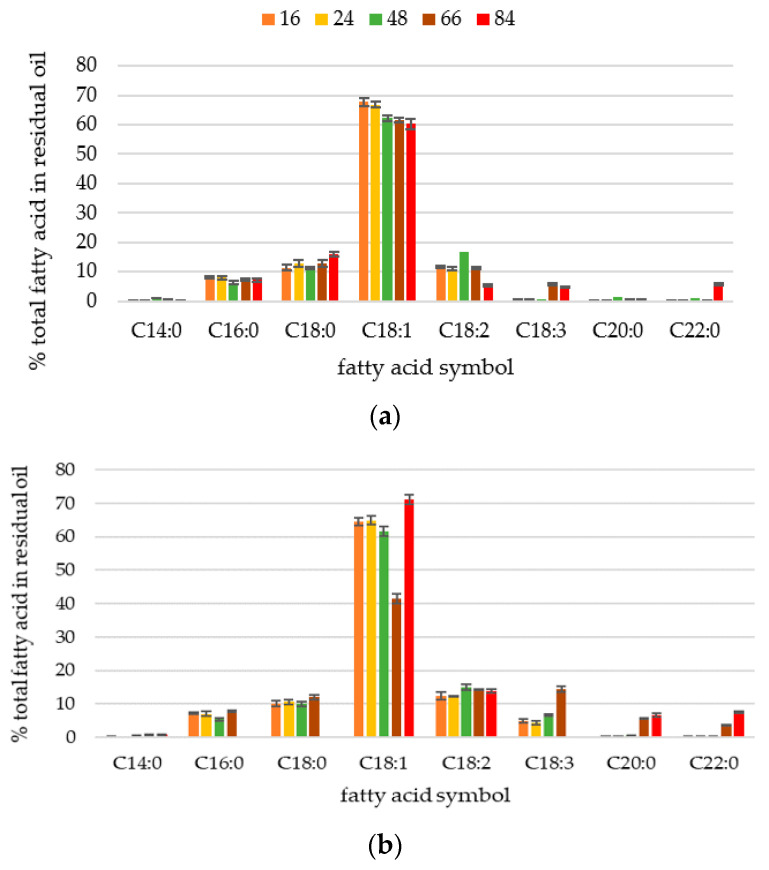
Profile of fatty acids in residual carbon source during fed-batch cultures of *Y. lipolytica* yeast cells grown in MO5 (**a**) and MO5-2xN (**b**) medium. The error bars indicate standard deviation.

The study aimed also to compare the changes in fatty acid content in residual oil during batch yeast cultures in two experimental media containing lipid carbon source (Figure 6). The results have further strengthened the confidence in the thesis that *Y. lipolytica* yeasts preferentially must accumulate unsaturated C18:1 and C18:2 fatty acids. The relation between the individual fatty acids did not change significantly during growth of cells. Moreover, the fatty acid profile of residual oil resembled the composition of cellular lipids in yeast (Figure 5).

### 2.2. Transcription Levels of Selected Genes Involved in Storage Lipid Synthesis

**Figure 7:**
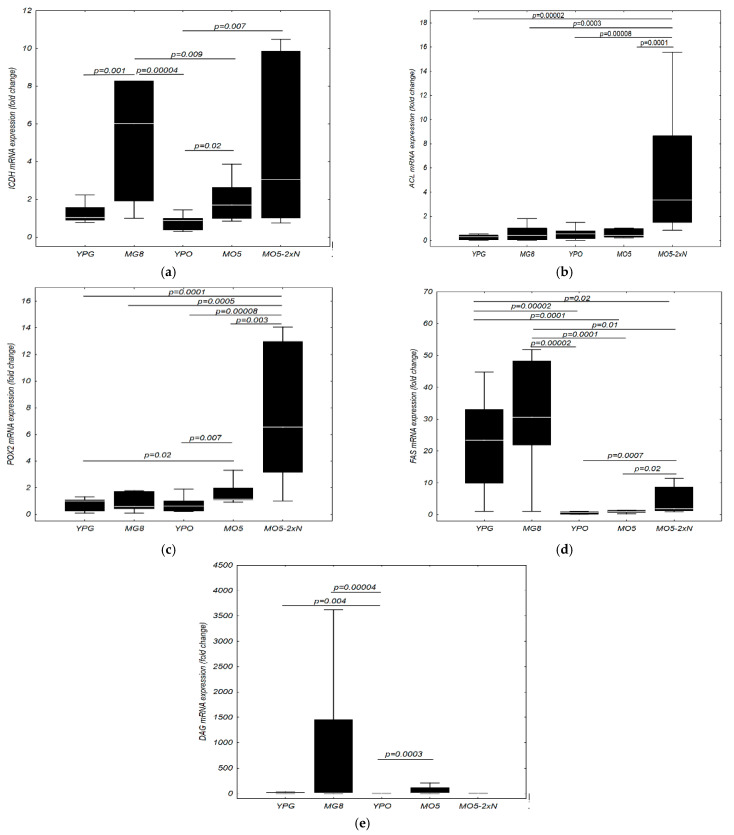
Average expression of the *ICDH* (**a**), *ACL* (**b**), *POX2* (**c**), *FAS* (**d**), *DAG* (**e**) mRNA in yeast cells of *Y. lipolytica* KKP 379 during batch cultures in control YPG and YPO medium, MG8 (containing glucose) and MO5 and MO5-2xN (containing olive oil) media during 84 h culture. The results are presented as the median (line), the percentile range (frame) and the range of minimum—maximum.

Environmental conditions make an undoubted difference in the mRNA transcription levels of genes, which can be also observed in the present study analysis of the mRNA expression of selected genes in subsequent growth phases. In the experiment, the mRNA expression of five genes of lipid catabolism and anabolism were analysed. The average expression of mRNA in yeast cells of *Y. lipolytica* during batch cultures was presented in Figure 7 and changes in gene expression during batch cultures are presented in Supplementary Figures S1–S5.

The 18s rRNA gene was selected as a reference gene, because it revealed the earliest and comparable CT mean values in all samples. We have chosen the gene whose expression was high in each cell and was characterized by fairly constant readings (similar CT means in samples with similar RNA concentrations meant that the gene’s expression was on a similar level in all samples) (Supplementary Table S1).

Among the transcripts, mRNA expression for the following enzymes was analysed: NAD-dependent isocitrate dehydrogenase (inhibited in the de novo pathway, encoded by *ICDH* gene), ATP-dependent citrate lyase (Acl, highly active in the de novo pathway, encoded by *ACL* gene), acyl-CoA oxidase II (Aox, with high activity in the ex novo pathway in the presence of fatty acids, encoded by the *POX2* gene), fatty acid synthase complex (with high de novo activity, encoded by *FAS* gene), diacylglycerol acyltransferase (active in both pathways, encoded by *DAG* gene) (Figure 1).

The level of *ACL* transcription was unexpectedly low contrary to the results of preliminary studies by Fabiszewska et al. [11] (Supplementary Figure S1). The most informative experiment occurred in batch cultures provided in MO5-2xN medium, when the mRNA expression of selected genes were relatively high. An also congruent profile of the mRNA expression changes were noticed for MO5 and YPO media. Higher mRNA expression was observed for the first two days and then, for the log phase duration, the fold change of mRNA decreased. Decisive growth of *ACL* mRNA expression could be seen in nitrogen-limited MG8 after 3 days of culture, probably as an effect of nitrogen starvation, what was not observed in MO5 and MO5-2xN medium. The increase in *ACL* mRNA expression occurred at 42 h and it was still ten times higher on the 3rd and 4th days in MO5-2xN medium in relation to the other four media. *ACL* mRNA sharply increased in MO5-2xN medium after 84 h and in YPO medium, suggesting that the increase in *ACL* gene expression is not due to nitrogen starvation. The *ACL* mRNA expression did not turn out to be a good marker of the de novo pathway of lipid biosynthesis for *Y. lipolytica* yeast as no evident differences could be seen between mRNA expression in glucose and olive oil containing media with rich or poor nitrogen source content.

The highest expression of the fatty acid synthase (*FAS*) mRNA was observed for cells grown in YPG and MG8 control media, which confirmed the thesis that this synthase was inhibited by free fatty acids present in lipid-containing media (Supplementary Figure S2). The response rate in YPG medium reached a maximum at 36 h (ca. 40 fold change) and in MG8 the fold change exceeded 100 at 60 h. We did not find changes in *FAS* mRNA expression in MO5 and YPO, as opposed to MO5-2xN medium where this expression was significantly higher; this, in turn, proved the possibility of partial inhibition of this enzyme in media with a lipid carbon source. Interestingly, the increase in *FAS* mRNA changes was detected on the fourth day of the experiment and was twice as high as the level of the mRNA expression in the early logarithmic phase.

In MO5-2xN medium, an increase in *FAS* mRNA expression coincided with the increase of *DAG* mRNA expression activity. Meanwhile, in MO5 and MG8 medium, the higher *FAS* mRNA expression occurred approximately several hours after the *DAG* mRNA expression increased (in the MO5 medium, FAS increased after 36 h and DAG increased after 20 h while, in the MG8 medium, FAS increase was observed after 60 h and DAG increase after 48 h). The positive correlation between the level of *DAG* transcription level and the content of microbial oil in the cell turned out to be extremely interesting (Supplementary Figure S3). For proper interpretation of the pathway of cell storage lipid accumulation, those two parameters should analysed together. In our previous work [12] we concluded that the maximum content of intracellular lipids was reached after ca. 2 days of culture (38 h, 0.46 g/g DM) in MO5 medium under similar bioreactor culture parameters (the high level of mRNA expression for DAG was observed from 20 to 42 h). After that time, it is probable that hydrolysis of triacylglycerols in lipid bodies occurred and fatty acids could be used as an energy source (Figure 1 and Figure 4), so at 84 h the content of lipids was only ca. 0.08 g/g DM). In MO5-2xN medium, biosynthesis of storage lipids was observed later and the sharp increase in *DAG* mRNA expression may stand as the evidence (Figure 4 and Figure S3). For MG7 medium [12] with a composition similar to MG8 medium, with the lower glucose content by 1%, the maximum conversion yield of biomass per carbon substrate was observed at 40 h (0.28 g/g DM). Based on those results, it can be summarized that also in a glucose-rich medium, the *DAG* mRNA expression followed maximum storage lipid biosynthesis. Nevertheless, the fold change of *DAG* mRNA expression was 30 to 40 times higher in MG8 medium in relation to MO5 medium. It seemed puzzling, in the context of lipid biosynthesis yield which occurred higher in olive oil media, why the *DAG* mRNA expression was so low.

**Figure 8:**
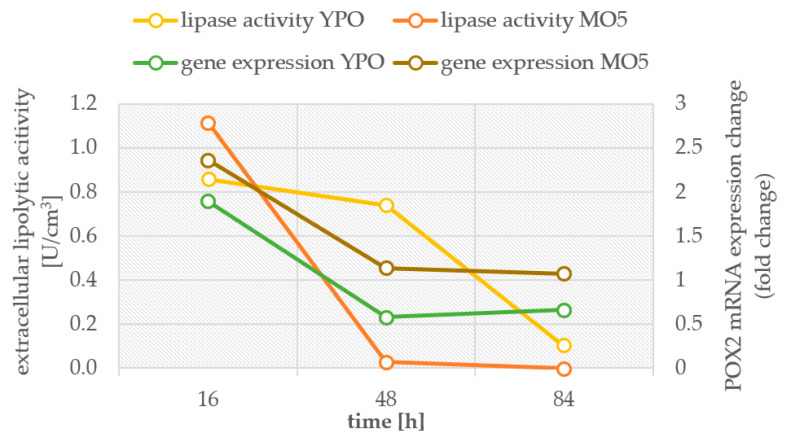
The correlation between *POX2* gene expression level and extracellular lipase activity during *Y. lipolytica* batch culture in control YPO medium and experimental nitrogen-limited MO5 medium.

It was observed that the level of changes for mRNA expression of genes encoding NAD-dependent isocitrate dehydrogenase (*ICDH*) (Supplementary Figure S4) and ATP-dependent citrate lyase (*ACL*) was significantly higher in the MO5-2xN medium, although in the case of the *ICDH* gene this level was comparable to the MG8 medium. Low mRNA expression was observed for control media. The highest mRNA expression of *POX2* was observed in the MO5 and MO5-2xN medium (Supplementary Figure S5). It is significant to note that a several times higher fold change of mRNA expression was observed when double nitrogen dose was applied in comparison to MO5 medium which can be explained by higher biomass yield and higher nitrogen dose in the medium and more intensive oxidation of fatty acids. Surprisingly, an increase in *POX2* mRNA expression was observed on the fourth day of culture also in MG8 medium. A significantly higher level of mRNA expression changes was demonstrated in the medium with a higher carbon to nitrogen ratio. The *POX2* transcription was correlated with extracellular lipase activity measured during batch cultures in control lipid-rich YPO medium and nitrogen limited MO5 medium (Figure 8). The straightforward correlation could be observed between high lipase activity and the mRNA expression change.

## 3. Discussion

Lipid accumulation as a reserve storage is triggered by an excess of carbon source and one limiting factor [13]. In the study, nitrogen source was the limiting nutrient. Under these conditions, the carbon flux is directly channelled toward lipid synthesis and discrete oil droplets consisting of triacylglycerols are formed within the cells [4,13]. Additionally, the stored lipids usually reach their highest levels at the start of the stationary phase when the oleaginous cells exhaust their nutrient supply and have excess carbon [14]. This is in good agreement with our observations of the course of lipid accumulation. In the lag and early log phases, when all nutrients are in excess, the biomass production occurs. When the nitrogen source is exhausted, cell division is reduced and the lipid storage begins. It could be also observed that the cellular lipid content in the various trials might have been higher before the end of culture due to intracellular lipid break-down which had been already demonstrated in *Y. lipolytica*, irrespective of carbon source used as a substrate [15,16].

The analysis of the fatty acid profile of lipids extracted from cells lends support to previous findings in literature; specifically, Yarrowia yeast accumulated lipids in a medium with plant oil is similar in composition to the initial substrate [12,17]. Nonetheless, some reports disagree with this, suggesting that cellular fatty acid composition can present very high differentiations compared to those obtained during growth on glucose [15,18].

The study aimed to investigate the molecular markers, which can be related to lipid accumulation in oleaginous yeast cells. mRNA expression of five genes of lipid catabolism and anabolism were studied in five culture media. Although ex novo synthesis of lipids may occur, regardless of whether nitrogen is available and is thought to occur simultaneously with cell growth [1], all experimental media lacked a nitrogen source. The choice was based on our preliminary studies when in nitrogen-rich media low lipid accumulation was observed [12]. Those observations stayed also in agreement with the hypothesis that in lipid-rich media some de novo pathway is responsible for high lipid content in the cell.

In order to analyse the selected gene expression, the reference gene should be chosen. There are not many reports regarding reference genes for *Y. lipolytica* real-time RT-PCR assays [19]. Among four most commonly studied, the 18 s rRNA have been chosen the most useful to normalize data on expression levels of some genes during yeast batch culture. Rzechonek et al. used actin as a reference gene when studying *EUF1*, a newly identified gene involved in erythritol utilization in *Y. lipolytica* [20]. Similarly, actin played a role of housekeeping gene in a study on interaction of the yeast in ecosystem with *Staphylococcus xylosus* and *Lactococcus lactis* and there was found that the *ACT* expression was unaffected by culture conditions in *Y. lipolytica* [21,22].

It should be mentioned that nitrogen exhaustion provokes the following: a release of ammonium ions from AMP, that alters the Krebs cycle function; isocitrate dehydrogenase activity (ICDH); and, as a consequence, an isocitrate and citrate concentration increase. Cytosolic citrate, CoA and ATP is converted into acetyl-CoA by Acl, ADP and oxaloacetate (Figure 1); additionally, it is claimed as a key enzyme in de novo route [1]. Dulermo et al. [6,10] proved that inactivation of *ACL* decreases fatty acid synthesis by 60 to 80%, confirming its essential role in lipid synthesis in *Y. lipolytica*. Surprisingly, based on our own experiments, the ATP-citrate lyase did not indicate being a good genetic marker for the de novo lipid biosynthesis pathway and the thesis on *ACL* inhibition by fatty acids could not be acknowledged. A reasonable explanation for this may be found when comparing changes in *ACL* gene expression during *Y. lipolytica* batch culture in lipid-rich media and in glucose-based media. There was evidence that the *ACL* mRNA expression level was independent of carbon source. Nevertheless, this is the key enzyme in the de novo route; due to nitrogen starvation the increasing concentration of citrate in mitochondria did not influence an increase of its expression. Both in YPO and MO5 media, in stationary growth phase, an unexpected increase in mRNA expression was noted. What can be outlined is the fact that the basic level of *ACL* mRNA expression was observed for all variants of cultures in control and experimental media.

The fatty acid synthase gene seemed to better distinguish two routes of lipid biosynthesis in oleaginous yeast cells. The correlation between *FAS* mRNA expression and kind of carbon source was relevant. High *FAS* mRNA expression levels for cells grown in YPG and MG8 control media confirmed the thesis that this synthase was inhibited by free fatty acids present in lipid-containing media that could be associated with a high content of acyl-CoA—a negative regulator of fatty acid synthase [23]. Moreover, depletion of lipid substrate in the medium seemed to unblock the mechanism and intensifying storage lipid accumulation in nitrogen-limited media. The reason for this rather contradictory result is still not completely clear. The yeast *Y. lipolytica* could perform de novo fatty acid biosynthesis from glucose in spite of the presence of exogenous long-chain fatty acids in the culture medium [1,24].

*Y. lipolytica* accumulates in lipid bodies mainly triacylglycerols (85%) formed through the Kennedy pathway, where diacylglycerol is converted into triacylglycerols from phospholipids or from acyl-CoA by diacylglycerol acyltransferase [25,26]. Strong evidence that *DAG* could be a good marker for monitoring the process of lipid storage in lipid bodies was found when cells were grown in lipid substrate medium (MO5 and MO5-2xN). Such a trend was not attributed to glucose-containing media.

Analysing the results led to the hypothesis that the sequence of metabolic processes could be proposed a little bit differently from that suggested so far, as presented in Figure 1. Free fatty acids that are a product of hydrolysis of triacylglycerols in lipid bodies are degraded in β-oxidation and the acetyl-CoA could be a substrate for a fatty acid synthase enzymatic complex. This is a hypothetical connection point, between the de novo and ex novo routes, which should be further verified. Lipid remobilization from lipid bodies to fatty acids activated and transported to peroxisome for carrying out β-oxidation is regulated in a complex way. Notably, alternative pathways of non-activated fatty acids are also known [7]. Clearly, whether acyl-CoA is used in the anabolic pathway (lipid biosynthesis) or the catabolic pathway (β-oxidation) determines the lipid content of the cells, which was also reviewed by Abgahri and Chen [8]. Shifting the balance to one side depends upon physiological and environmental conditions and is regulated based on energy intake and expenditure [27]. The general regulatory mechanism of lipid accumulation in the oleaginous microorganisms has not yet been elucidated but the results of the present study confirmed that the flux through lipid pathway is correlated to specific yeast growth phases and to the complex models of intermediate transport between different organelles. As reviewed by Papanikolaou and Aggelis [1], it has been demonstrated in many investigations that the oleaginous microorganisms do not possess a hyperactive system of fatty acid biosynthesis, but they produce in significant quantities, acetyl-CoA as a building block for lipid molecules.

In discussing the *POX2* gene expression observations, it is interesting to note that the sharp decrease in *DAG* expression at the end of the second culture day in MO5 medium corresponded to increased *POX2* gene expression on the third day. The high levels of their mRNA expression were significant on the first day of the experiment when the early log phase lasted. This is in good agreement with other authors who observed that a large battery of extracellular lipases produce fatty acids out of oils, which will be rapidly incorporated into the cell [3]. Lipid bodies represent a dynamic reserve carbon source and under depleting nutrient conditions storage triacylglycerols hydrolysis occurs. Fatty acid degradation provides energy and starts by fatty acid oxidation catalysed by acetyl-CoA oxidases (Aoxs)*. POX2* gene encodes for long-chain specific Aox and five others *POX* family genes are described [28].

The mitochondrial enzyme isocitrate dehydrogenase works with NAD to catalyse the oxidation of isocitrate to α-ketoglutarate. The enzyme is inhibited by NADH and ATP. When the energy level in the cell is low (low amount of ATP and NADH), the Krebs cycle is faster and reduced NADH is needed for the oxaloacetate to malate conversion reaction [29]. In MG8 and MO5-2xN, significantly higher expression levels were observed for the *ICDH* gene. Regrettably, this issue could not be explained fully. What is important, from acetyl-CoA, two carbon atoms are introduced into the Krebs cycle in a reaction catalysed by the product of the *ICDH* gene and α-ketoglutarate dehydrogenase. Hence, in the MG8 medium, the high activity of the gene encoding the enzyme was compared to the low levels observed in the MO5 medium, where carbon from acetyl-CoA was used for the synthesis of oxaloacetate in the glyoxal cycle (Figure 1). Hypothetically, *ICDH* gene could be inhibited by succinate, which may be the subject of in-depth metabolic studies in the future.

It is worth emphasizing that there was a significant difference between the MO5 and MO5-2xN media in the context of the expression level of *ICDH* mRNA. Presumably, due to a twice lower C/N ratio and a higher nitrogen dose in the MO5-2xN medium, the cells were characterized by a higher demand for energy, and moreover, the Krebs cycle was inhibited to a much lesser extent than in the MO5 medium. The explanation for this may be related to the results found by other authors [1,30] who claimed that isocitrate dehydrogenase show a very limited activity during lipid-accumulating conditions.

## 4. Materials and Methods

### 4.1. Yeast Strain and Culture Conditions

The *Y. lipolytica* KKP 379 wild-type strain was purchased from the Collection of Industrial Microorganisms at the Prof. Wacław Dąbrowski Institute of Agricultural and Food Biotechnology-State Research Institute in Warsaw, Poland. The strain was stored in 20% (*v*/*v*) glycerol solution in nutrient YPG broth (yeast extract 1%, peptone 2%, glucose 2%) at −20 °C.

Inoculum pre-culture was grown in YPG medium on a rotary shaker at 28 °C for 24 h. Number of yeast cells in inoculum pre-culture was determined by plate method on YPG agar medium and it ranged from 8.0 to 8.5 log CFU/cm^3^ (average 8.29 ± 0.21 log CFU/cm^3^). Rich medium YPG (containing 2% *w*/*v* glucose) and YPO (containing 2% *w*/*v* olive oil) were used as a control during batch bioreactor cultures and mineral media MO5 and MO5-2xN (containing 5% *w*/*v* olive oil) and MG8 (containing 8% *w*/*v* glucose) were used as media stimulating storage lipid synthesis. The detailed composition of media are shown in Table 1.

All inorganic salts and glucose were purchased from Avantor Performance Materials Poland S.A. (Gliwice, Poland), yeast extract and peptone were obtained from BTL (Łódź, Poland); olive oil was purchased from local market (Aceites Borges Pont, Terrega, Spain). Cultivation was typically performed as follows. Initial preculture was performed on YPG broth on a rotary shaker at 28 °C, 140 rpm overnight. The experimental cultures were provided in a BIO FLO 3000 bioreactor (New Brunswick, Hamburg, Germany) for 84 h at 28 °C, 350 rpm agitator speed and 0.025% (*v*/*v*) inoculum as described by Fabiszewska et al. [11].

### 4.2. General Analytical Techniques

Standard microbiological methods were used throughout this study. Growth phases of yeast were evaluated on the basis of changes in the level of relative medium oxygenation and biomass yield. The culture medium pH was measured by pH electrode. Biomass was centrifuged and washed with hexane in order to eliminate the fat traces from yeast biomass. Biomass yield was evaluated by drying at 105 °C until constant weight. Glucose content was measured by the 3,5-dinitrosalicylic acid (DNS) colorimetric method at 540 nm in which 3,5-DNS in alkaline solution is reduced to 3-amino-5-nitrosalicylic acid by reducing saccharide molecules. Standard curve of glucose was prepared and used to estimate the concentration of the unknown reducing saccharide concentration. Residual oil in medium and cellular lipid concentrations were determined by extraction-weight method with twice extraction of lipids by hexane. Intracellular lipids were extracted by leaching in Soxhlet extractor in case of yeast cells with hexane. Fatty acid composition was evaluated by gas chromatography according to the parameters established in the past works. Fatty acids derivatization was provided using 1 M sodium methoxide and 10% BF3. FAMEs (fatty acids methyl esters) were extracted with 4 mL of n-hexane. Hexane phase was analyzed with GC-FID. Methyl heptadecanoate was used as an internal standard. An apparatus from Agilent Technologies 68790 N GC (Santa Clara, CA, USA) equipped with a capillary column HP 5-MS was used for the analysis (column parameters: 0.25 mm internal diameter and 0.25 μm stationary phase film thickness). Helium was used as the carrier gas. Individual fatty acids were identified on the basis of retention times, comparing them with reference ones [11,12].

### 4.3. Lipase Activity Assay

Measures of enzymatic activity were carried out using the spectrophotometric method based on the hydrolysis of *p*-nitrophenyl laurate [31]. Reactions were carried out in Erlenmeyer flasks with vigorous stirring for 30 min at 37 °C. Of the substrate, 0.3 Mmol was suspended in 2 cm^3^ of heptane. *p*-nitrophenyl laurate was added to 1 cm^3^ of supernatant and buffered at pH 7.0 to the volume 15 cm^3^. One unit of enzyme activity was defined as the enzyme quantity that liberated 1 µmol of *p*-nitrophenol per minute under the assay conditions at 37 °C. For the reaction, *p*-nitrophenyl laurate was used, the lipase has a specificity towards insoluble or at least poorly soluble long-chain fatty acids in contrast to esterase which activity is found to be highest towards more water soluble substrates [32].

### 4.4. Quantification of Genes Expression

Total RNA was isolated according to method by Chomczyński et al. [33]. The reverse transcription was made using cDNA Synthesis Kit with dsDNase (Thermo Fisher, Waltham, MA, USA). Real time-PCR was performed in Prism 7500 (Applied Biosystems, Foster City, CA, USA) according to the procedure described by Livak et al. [34]. The PCR conditions were: 10 min 25 °C; 20 min 50 °C; 5 min 85 °C. Four genes were verified as endogenous control genes (*ACT1* gene encoding actin, YALI0D08272g, *β1* gene encoding β—tubulin, YALI0E26961p, *GAPDH* gene encoding glyceraldehyde-3-phosphate dehydrogenase, YALI0C06369g and eukaryotic 18s rRNA gene) of which 18srRNA was selected as the best endogenous control (Supplementary Table S1) to normalize the level of the tested genes (*ACL* gene encoding ATP-citrate lyase subunit 1, YALI0E34793p; *ICDH* gene encoding NADP-dependent isocitrate dehydrogenase, YALI0_F04095g; *FAS* gene encoding fatty acid synthase subunit beta, YALI0B15059g; *DAG* gene encoding diacylglycerol acyltransferase, YALI0C06369g and *POX2* gene encoding acyl-coenzyme A oxidase 2, YALI0_F10857g).

Sequences for primers and probes:

***ACL***

FW: TTC CGA ACC ATC GCC ATT A

REV: TGG GCC TTG TGG AGG ATC T

PROBE: CCC GAG CGA CGA GCC CGA

***ICDH***

FW: CGTGCAGTCCGACATTGTTG

REV: GGTGACGAGAACAGAGGTCATG

PROBE: CCAGGGCTTTGGCTCTCTCGGTC

***FAS***

FW: AAAGCCGATGGATCGATGTC

REV: GGAAAAGCTCTGCAACACAGAA

PROBE: CTCCGAAATCTTGCCGGCACTTTCAT

***DAG***

FW: TCCGCCGTCATCCATGA

REV: GGCGGCTCCGATGATGT

PROBE: CTGCTTGTCGGCATCCCCACTCA

***POX2***

FW: GAT GGA TCC AGT TCA CCA ACG T

REV: GTT ACC CTC TCG GTC GAC CTT T

PROBE: CCC CCG ACA GAA CCT GCT CAT GAA

***ACT1***

FW: CGAGCGAATGCACAAGGA

REV: GAGCGGTGATCTTGACCTTGA

PROBE: ATCTCCGCTCTTGCCCCCACCTC

***β1***

FW: CTCGGTTCTTCCCTCACCTAAG

REV: GGTGGAAAGAGAGCACAGCAT

PROBE: TCTCCGAGACCGTCACTGAGCCCT

***GAPDH***

FW: CGAGGTCGTCGCTGTGAAC

REV: GGGTGGAGTCGTACTTGAACATG

PROBE: CCTTCATCGACACCGAGTACGCTGCTT

**18s rRNA:** Eukaryotic 18S rRNA Endogenous Control (Hs99999901_s1)

The nucleotide sequences were identified using the BLAST search at NCBI.

### 4.5. Statistical Analysis

Statistical analyses were performed using Statistica 13.1 software (TIBCO Software Inc., Palo Alto, CA, USA). *p*-value lower than 0.05 was considered to be statistically significant. The results obtained in the CT (cycle threshold) values from real time-qPCR experiments were analysed by U Mann–Whitney test. The Shapiro–Wilk test was used for the normality of data distribution. ANOVA analysis and Tukey’s multiple comparison test were applied for other data comparisons. Cultures were provided in duplicates.

## 5. Conclusions

Taken as a whole, our observations once again did not exclude the possible coexistence of two biochemical routes resulting in triacylglycerol synthesis in oleaginous microorganism cells. Our study provided additional support for the thesis that we should speak about the advantage of one biochemical pathway over another, de novo or ex novo, rather than about choosing a single route by the cell depending on carbon source present in a culture medium (olive oil or glucose) and nitrogen starvation conditions. The processes of lipid synthesis and storage and lipid hydrolysis and oxidation for energy purposes are tightly interdependent. Thus, there are grounds for claiming a dynamic mobility of the cell which is able to switch on or off these two opposite biochemical routes and for exploring this claim in future perspectives.

## Figures and Tables

**Table 1:** Culture media in bioreactor experiments.

Culture Medium	Carbon Source [g/dm^3^]	Inorganic Nitrogen Source (NH_4_)_2_SO_4_ [g/dm^3^]	Medium Composition [g/dm^3^]
YPG	glucose, 20.0	-	yeast extract, 10.0; peptone, 20.0
YPO	olive oil, 20.0	-	
MO5	olive oil, 50.0	2.5	KH_2_PO_4_, 7.0; Na_2_HPO_4_, 2.5; FeSO_4_ x H_2_O, 0.16; CaCl_2_, 0.15; MnCl_2_ x 4H_2_O, 0.08; ZnSO_4_, 0.02; yeast extract, 2.0; peptone, 1.0
MO5x2N	olive oil, 50.0	5.0	
MG8	Glucose, 80.0	2.5	

## Supplementary Materials

The following are available online at https://www.mdpi.com/article/10.3390/ijms23031041/s1.
