# Supplementary material for: Expression Profile of Selected Genes Involved in Storage Lipid Synthesis in a Model Oleaginous Yeast Species Yarrowia lipolytica
Source: Int J Mol Sci. 2022 Jan 18;23(3):1041. doi: 10.3390/ijms23031041 (PMC8834811; doi:10.3390/ijms23031041)
Supplement: Supplementary file 1 [file ijms-23-01041-s001.zip › ijms-1557126-supplementary.pdf]

Supplementary Material

Expression profile of selected genes involved in storage lipids synthesis in a model oleaginous yeast species *Yarrowia lipolytica*

Agata Fabiszewska [1,*], Magdalena Paplińska-Goryca [2], Paulina Misiukiewicz-Stępień [3], Małgorzata Wołoszynowska [4], Dorota Nowak [5] and Bartłomiej Zieniuk [1]

1 Department of Chemistry, Institute of Food Sciences, Warsaw University of Life Sciences-SGGW, 159c Nowoursynowska Street, 02-776 Warsaw, Poland; agata_fabiszewska@sggw.edu.pl (A.F.), bartlomiej_zieniuk@sggw.edu.pl (B.Z.);

2 Department of Internal Medicine, Pulmonary Diseases and Allergy, Medical University of Warsaw, Poland; mpaplinska@wum.edu.pl

3 Postgraduate School of Molecular Medicine, Medical University of Warsaw, 2a Trojdena Street, 02-091 Warsaw, Poland; pmisiukiewicz@wum.edu.pl

4 Łukasiewicz Research Network—Institute of Industrial Organic Chemistry, 6 Annopol Street, 03-236 Warsaw, Poland; malgorzata.woloszynowska@ipo.lukasiewicz.gov.pl

5 Department of Food Engineering and Process Management, Institute of Food Sciences, Warsaw University of Life Sciences-SGGW, Nowoursynowska Street 159c, 02-776 Warsaw, Poland; dorota_nowak@sggw.edu.pl

* Correspondence: agata_fabiszewska@sggw.edu.pl; Tel.: +48-22-59-37-621

Table S1. CT means for five reference genes tested in the study in yeast samples taken from MG8 and MO5 media during fed-batch cultures of *Y. lipolytica* 379. SD – standard deviation

Medium	Time	Reference gene name	CT mean	CT SD
MG8	16	18S rRNA	15.86255	0.152056
MG8	20	18S rRNA	13.24002	0.049804
MG8	24	18S rRNA	18.77367	0.197089
MG8	36	18S rRNA	16.22346	0.088201
MG8	42	18S rRNA	17.58364	0.070762
MG8	48	18S rRNA	20.06084	0.123887
MO5	16	18S rRNA	13.55049	0.01705
MO5	20	18S rRNA	16.91484	0.082929
MO5	24	18S rRNA	15.70398	0.079527
MO5	36	18S rRNA	17.28682	0.117434
MO5	42	18S rRNA	17.50926	0.013554
MO5	48	18S rRNA	17.60471	0.213186
MG8	16	18S rRNA	15.86255	0.152056
MG8	20	18S rRNA	13.24002	0.049804
MG8	24	18S rRNA	18.77367	0.197089
MG8	36	18S rRNA	16.22346	0.088201
MG8	42	18S rRNA	17.58364	0.070762
MG8	48	18S rRNA	20.06084	0.123887
MO5	16	18S rRNA	13.55049	0.01705
MO5	20	18S rRNA	16.91484	0.082929
MO5	24	18S rRNA	15.70398	0.079527
MO5	36	18S rRNA	17.28682	0.117434
MO5	42	18S rRNA	17.50926	0.013554
MO5	48	18S rRNA	17.60471	0.213186
MG8	16	actin	30.53583	1.131406
MG8	20	actin	31.47684	0.485092
MG8	24	actin	38.9252	0.840431
MG8	36	actin	36.20623	1.060652
MG8	42	actin	36.13245	2.299165
MG8	48	actin	37.83736	
MO5	16	actin	30.26344	0.141627
MO5	20	actin	32.81502	1.593832
MO5	24	actin	29.75557	0.976381
MO5	36	actin	28.26622	0.680439
MO5	42	actin	26.87859	0.399076
MO5	48	actin	26.37432	0.233538
MG8	16	actin	30.53583	1.131406
MG8	20	actin	31.47684	0.485092
MG8	24	actin	38.9252	0.840431
MG8	36	actin	36.20623	1.060652
MG8	42	actin	36.13245	2.299165
MG8	48	actin	37.83736	
MO5	16	actin	30.26344	0.141627
MO5	20	actin	32.81502	1.593832
MO5	24	actin	29.75557	0.976381

MO5	36	actin	28.26622	0.680439
MO5	42	actin	26.87859	0.399076
MO5	48	actin	26.37432	0.233538
MG8	16	GAPDH	21.39776	0.188881
MG8	20	GAPDH	21.24527	0.598064
MG8	24	GAPDH	25.57069	0.23242
MG8	36	GAPDH	24.41174	0.070696
MG8	42	GAPDH	24.31777	0.174701
MG8	48	GAPDH	26.06961	0.160677
MO5	16	GAPDH	23.37892	0.191448
MO5	20	GAPDH	26.69224	0.001888
MO5	24	GAPDH	25.70122	0.22704
MO5	36	GAPDH	26.15969	0.044209
MO5	42	GAPDH	26.39577	0.116278
MO5	48	GAPDH	26.57144	0.137101
MG8	16	GAPDH	21.39776	0.188881
MG8	20	GAPDH	21.24527	0.598064
MG8	24	GAPDH	25.57069	0.23242
MG8	36	GAPDH	24.41174	0.070696
MG8	42	GAPDH	24.31777	0.174701
MG8	48	GAPDH	26.06961	0.160677
MO5	16	GAPDH	23.37892	0.191448
MO5	20	GAPDH	26.69224	0.001888
MO5	24	GAPDH	25.70122	0.22704
MO5	36	GAPDH	26.15969	0.044209
MO5	42	GAPDH	26.39577	0.116278
MO5	48	GAPDH	26.57144	0.137101
MG8	16	β -Tubulin	28.70368	0.484375
MG8	20	β -Tubulin	27.99236	0.113129
MG8	24	β -Tubulin	33.63328	0.29567
MG8	36	β -Tubulin	33.00349	0.017352
MG8	42	β -Tubulin	32.56603	0.014248
MG8	48	β -Tubulin	34.63532	0.28566
MO5	16	β -Tubulin	31.01873	0.16399
MO5	20	β -Tubulin	33.87304	0.271482
MO5	24	β -Tubulin	32.2142	0.327495
MO5	36	β -Tubulin	31.21967	0.231025
MO5	42	β -Tubulin	31.29364	0.217588
MO5	48	β -Tubulin	31.54985	0.008897
MG8	16	β -Tubulin	28.70368	0.484375
MG8	20	β -Tubulin	27.99236	0.113129
MG8	24	β -Tubulin	33.63328	0.29567
MG8	36	β -Tubulin	33.00349	0.017352
MG8	42	β -Tubulin	32.56603	0.014248
MG8	48	β -Tubulin	34.63532	0.28566
MO5	16	β -Tubulin	31.01873	0.16399
MO5	20	β -Tubulin	33.87304	0.271482
MO5	24	β -Tubulin	32.2142	0.327495
MO5	36	β -Tubulin	31.21967	0.231025
MO5	42	β -Tubulin	31.29364	0.217588
MO5	48	β -Tubulin	31.54985	0.008897

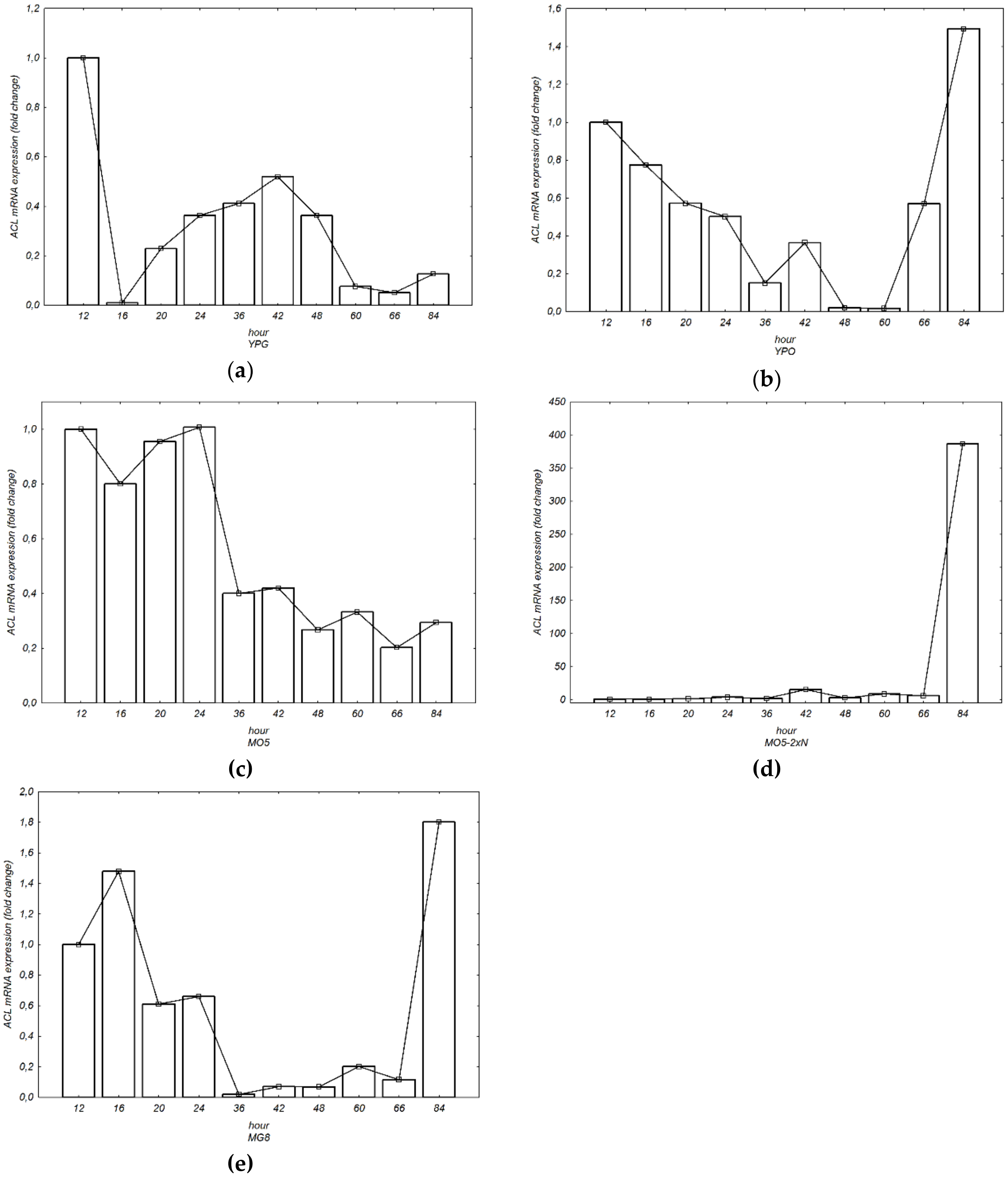

Figure S1. Changes in *ACL* mRNA expression during *Y. lipolytica* batch bioreactor culture in control YPG medium containing glucose (a), control YPO medium containing olive oil (b), MO5 (c); MO5-2xN (d) and MG8 (e) medium.

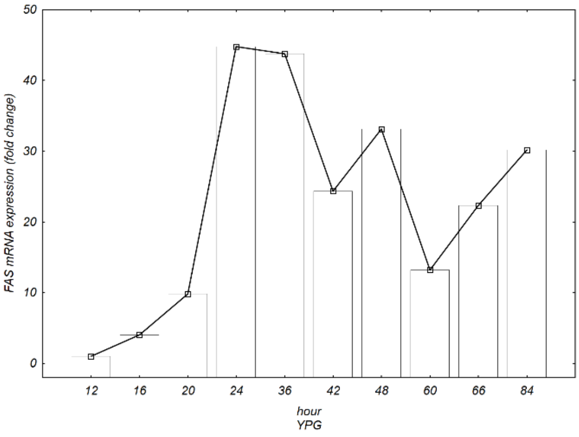

(**a**)

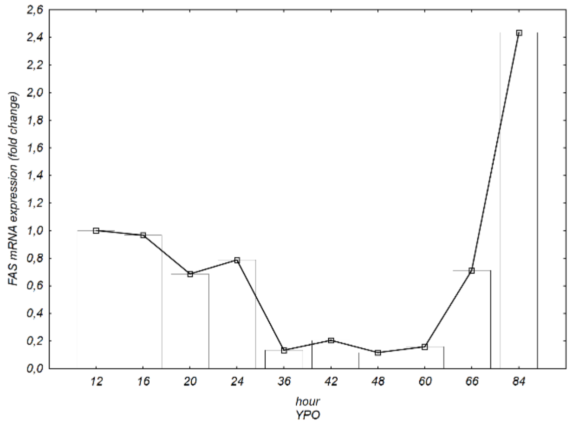

(**b**)

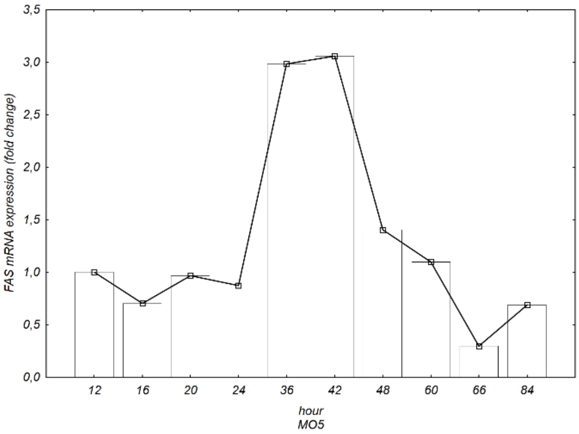

(c)

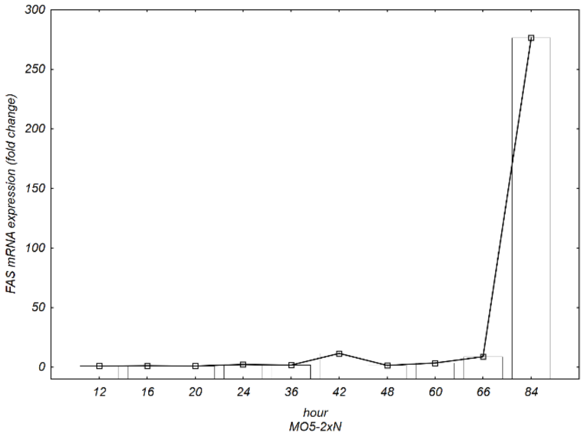

(d)

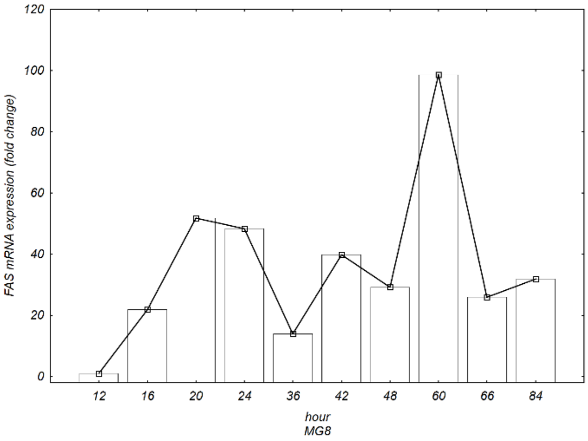

(e)

Figure S2. Changes in *FAS* mRNA expression during *Y. lipolytica* batch bioreactor culture in control YPG medium containing glucose (a), control YPO medium containing olive oil (b), MO5 (c); MO5-2xN (d) and MG8 (e) medium.

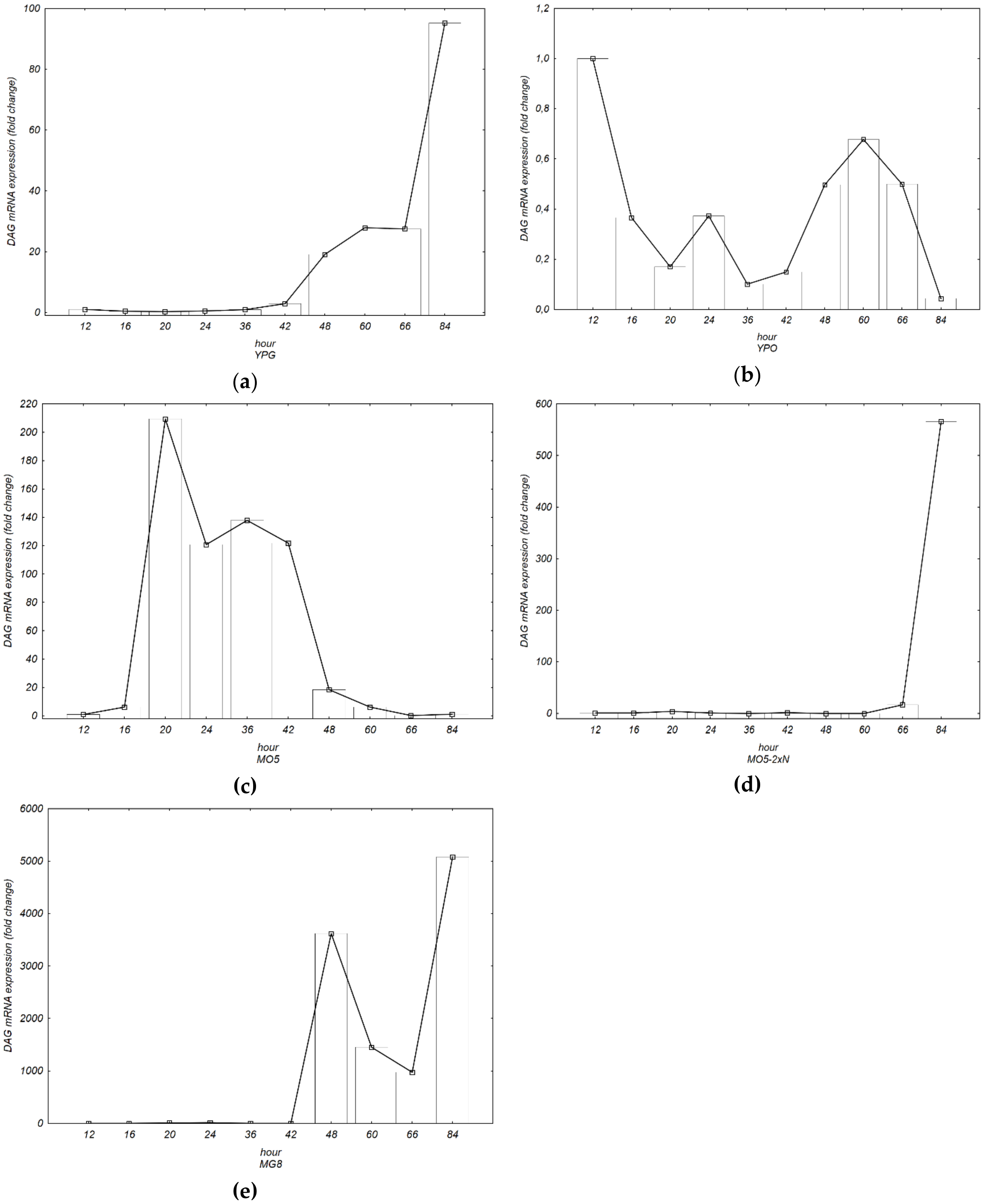

Figure S3. Changes in *DAG* mRNA expression during *Y. lipolytica* batch bioreactor culture in control YPG medium containing glucose (a), control YPO medium containing olive oil (b), MO5 (c); MO5-2xN (d) and MG8 (e) medium.

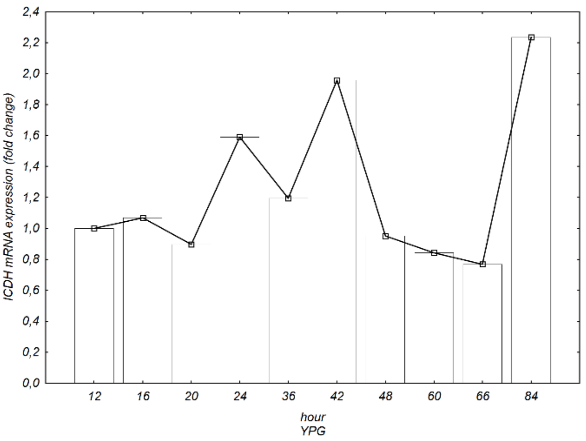

(**a**)

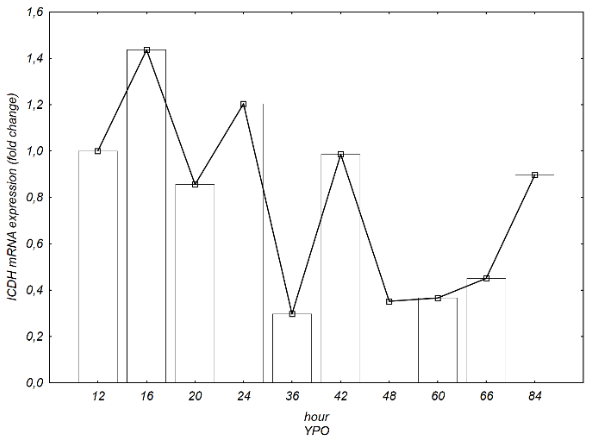

(**b**)

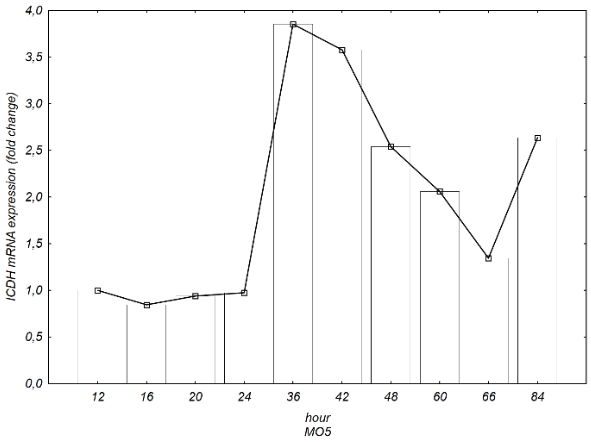

(**c**)

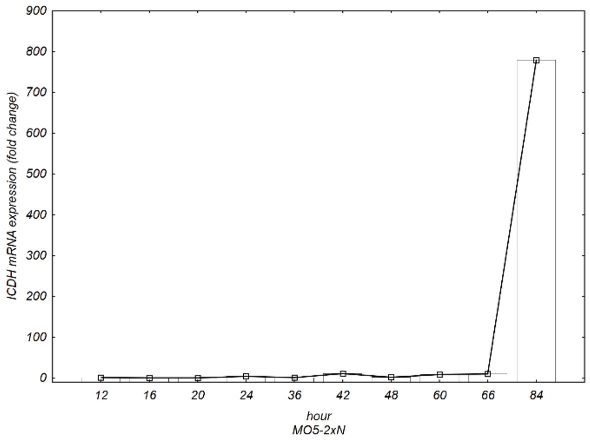

(**d**)

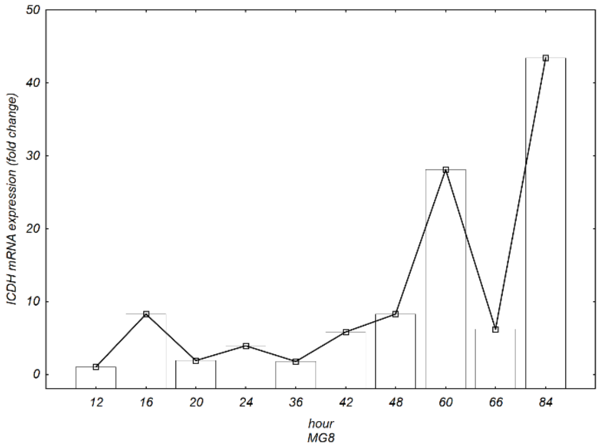

(**e**)

Figure S4. Changes in *ICDH* mRNA expression during *Y. lipolytica* batch bioreactor culture in control YPG medium containing glucose (a), control YPO medium containing olive oil (b), MO5 (c); MO5-2xN (d) and MG8 (e) medium.

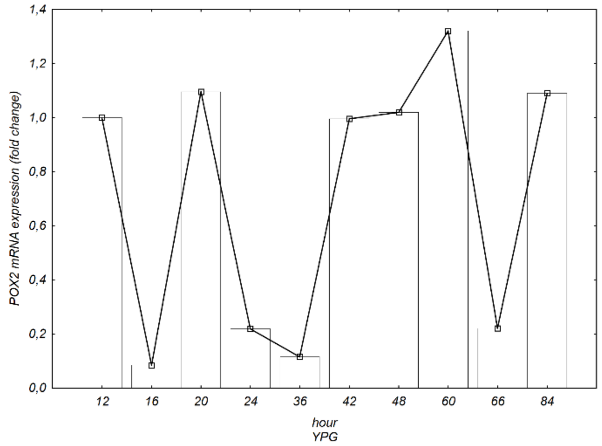

(**a**)

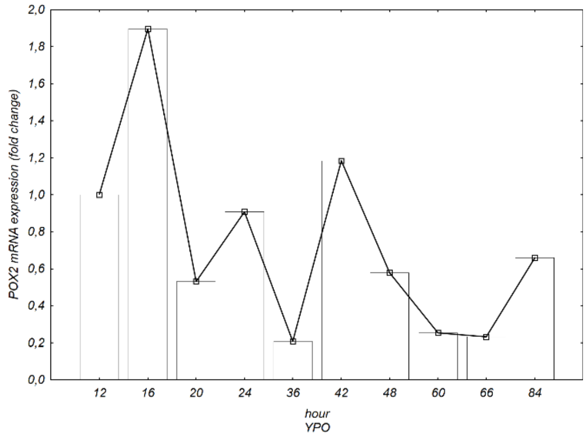

(**b**)

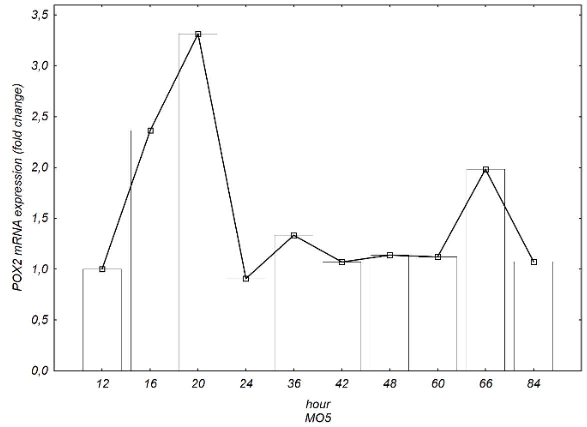

(**c**)

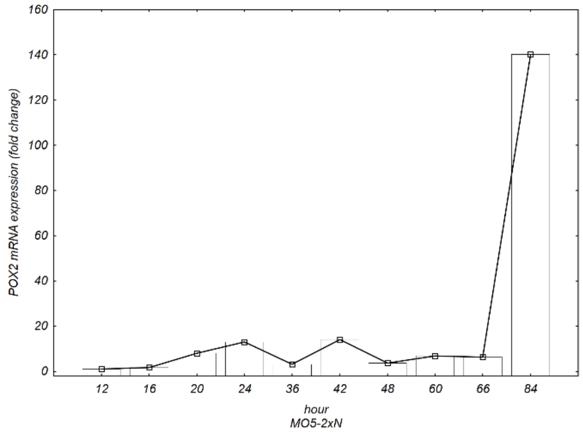

(**d**)

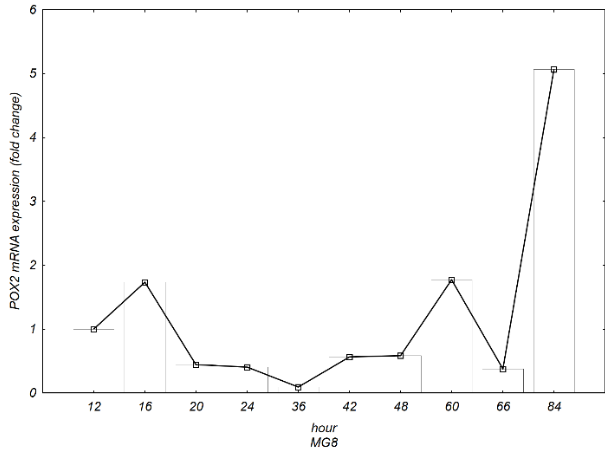

(**e**)

Figure S5. Changes in *POX2* mRNA expression during *Y. lipolytica* batch bioreactor culture in control YPG medium containing glucose (a), control YPO medium containing olive oil (b), MO5 (c); MO5-2xN (d) and MG8 (e) medium.
